# Genotyping-by-Sequencing of the regional Pacific abalone (*Haliotis discus*) genomes reveals population structures and patterns of gene flow

**DOI:** 10.1371/journal.pone.0247815

**Published:** 2021-04-07

**Authors:** Bo-Hye Nam, Hyaekang Kim, Donghyeok Seol, Heebal Kim, Eun Soo Noh, Eun Mi Kim, Jae Koo Noh, Young-Ok Kim, Jung Youn Park, Woori Kwak

**Affiliations:** 1 Biotechnology Research Division, National Institute of Fisheries Science, Busan, Republic of Korea; 2 Department of Agricultural Biotechnology and Research Institute of Agriculture and Life Sciences, Seoul National University, Seoul, Republic of Korea; 3 Genome, Inc, Seoul, Republic of Korea; University of California, UNITED STATES

## Abstract

Continuous monitoring of the present genetic status is essential to preserve the genetic resource of wild populations. In this study, we sequenced regional Pacific abalone *Haliotis discus* samples from three different locations around the Korean peninsula to assess population structure, utilizing Genotyping-by-Sequencing (GBS) method. Using *Pst*I enzyme for genome reduction, we demonstrated the resultant library represented the whole genome region with even spacing, and as a result 16,603 single nucleotide variants (SNVs) were produced. Genetic diversity and population structure were investigated using several methods, and a strong genetic heterogeneity was observed in the Korean abalone populations. Additionally, by comparison of the variant sets among population groups, we were able to discover 26 Korean abalone population-specific SNVs, potentially associated with phenotype differences. This is the first study demonstrating the feasibility of GBS for population genetic study on *H*. *discus*. Our results will provide valuable data for the genetic conservation and management of wild abalone populations in Korea and help future GBS studies on the marine mollusks.

## Introduction

Abalones are a type of marine gastropods belonging to the family of reef-dwelling snails, Haliotidae. There are about 70 different species distributed across tropical and temperate coastal areas, such as Australia, the United States, or East Asia. Abalones have been a valuable food source for humans worldwide, and they are also an important research resource for ecological and evolutionary studies due to their diverse phenotypic appearances and global distribution [1]. Among abalone species, the Pacific abalone *Haliotis discus* is known as the most popular and highly valued species for commercial fisheries resources owing to the quality of meat and traditional consumer preferences [2, 3]. The *H*. *discus* species has a wide geographic distribution in coastal waters of East Asia, and two subspecies exist: *H*. *discus hannai* inhabiting throughout Korea and northeastern Japan, and *H*. *discus discus* is distributed in the southern coastal areas of Korea and southwestern Japan [4].

For appropriate conservation of wild animals, continuous monitoring of the population structure and genetic status is essential because the maintenance of genetic diversity is important for whole species protection by providing disease- or stress-resistance genes to the gene pool [5]. Therefore, through genetic monitoring and biodiversity studies of wild populations, understanding the unique characteristics of regional populations and applying proper management strategies are suggested to preserve the genetic variation [6]. Previously, most studies on the Pacific abalones were, however, mainly focused on the development of artificial breeding programs and culture techniques such as seed production, spawning, or juvenile nursing, and there exist a relatively low number of researches carried out the genetic analysis of the wild populations [7, 8]. Moreover, these population genetics studies have been only performed using molecular markers such as microsatellites or mitochondrial DNA markers [9–11]. Most of them were carried out to mainly compare genetic diversity between wild and hatchery populations [12, 13]. So far, only two studies, utilizing microsatellite markers, explored genetic variation among regional wild abalone populations around the Korean peninsula. Traditionally, these molecular markers have been widely used for genetic studies of natural populations, even though it relied on small numbers of loci to make inferences [14]. In a few studies, those markers displayed lacked power to detect differentiation because they were limited to the narrow regions of the genome [15, 16]. For example, in a study using wild *Esox lucius* (Pike), restriction site-associated DNA sequencing (RAD-seq) not only resolved population genetic structure with better resolution than microsatellites but also were able to identify candidate loci under selection [17], and in another study, microsatellites had insufficient power to reveal the admixed status of one of the chicken populations in comparison with SNP dataset [18]. Thus, it could lead to insufficient and inconsistent results in abalones as well, making it difficult to simplify population characteristics and their evolutionary history [15]. Of the two population studies on regional Korean abalones mentioned above, one indicated genetic differentiation among populations collected from east, west, and southern coast [4], the other observed genetic separation between the eastern and pooled western and southern populations [19].

The recent advances in sequencing technology have led to enable sampling of the genome more densely and efficient production of more accurate and sufficient genetic information [15, 20]. One of the major recent advances has been the development of Genotyping-by-Sequencing (GBS). GBS uses restriction enzymes (REs) to reduce genome complexity and performs next-generation sequencing. The reduced representation approaches offer the ability to not only produce data covering the whole genome range with reduced cost but also provide high-resolution genetic information [21]. Furthermore, GBS can be employed for organisms with little or no previous genomic information [22]. With these advantages, it can provide major benefits especially in ecological and conservation genomics, since it is usually desirable to have a large sample size for studying wild populations, and sequencing of these samples with large genomes can cost a lot, particularly where the reference sequence information is absent [22]. GBS was originally designed for SNP genotyping in plant species, but recently the protocol has been quickly adopted to a wide variety of species, including marine animals [15]. For mollusks, GBS method has been applied in the construction of genetic linkage map of *Ruditapes philippinarum* (manila clam), the genomic best linear unbiased prediction (GBLUP) study of *Perna canaliculus* (greenshell mussel), and the population studies of *Haliotis fulgens* and *Haliotis laevigata* along the Western Australian coast [23–26]. Yet, it had not been previously utilized for research on *H*. *discus*.

In this present study, we performed GBS in a total of 102 regional abalones consisting of three regional Pacific abalone populations (*H*. *discus hannai*) collected from each of three seas (East, West, and South) in Korea and two outgroup populations, one Japan population (*H*. *discus discus*) and one red abalone population (*H*. *rufescens*). Using the variant information, we evaluated the genetic diversity and the population structure of *H*. *discus* populations inhabiting around the Korean peninsula and elucidated the impacts of geographical features and ocean currents in the gene flow among marine mollusks. Furthermore, by comparison of variant sets against red abalones, morphologically very different from *H*. *discus*, Korean Pacific abalone population-specific variants were discovered, which may be implicated with phenotypically different traits between groups. The results of the present study will provide an assessment of the utility of GBS for genetic analysis of abalone species and contribute to the management and genetic conservation of the wild Pacific abalone populations.

## Materials and methods

### Sample collection

**Fig 1:**
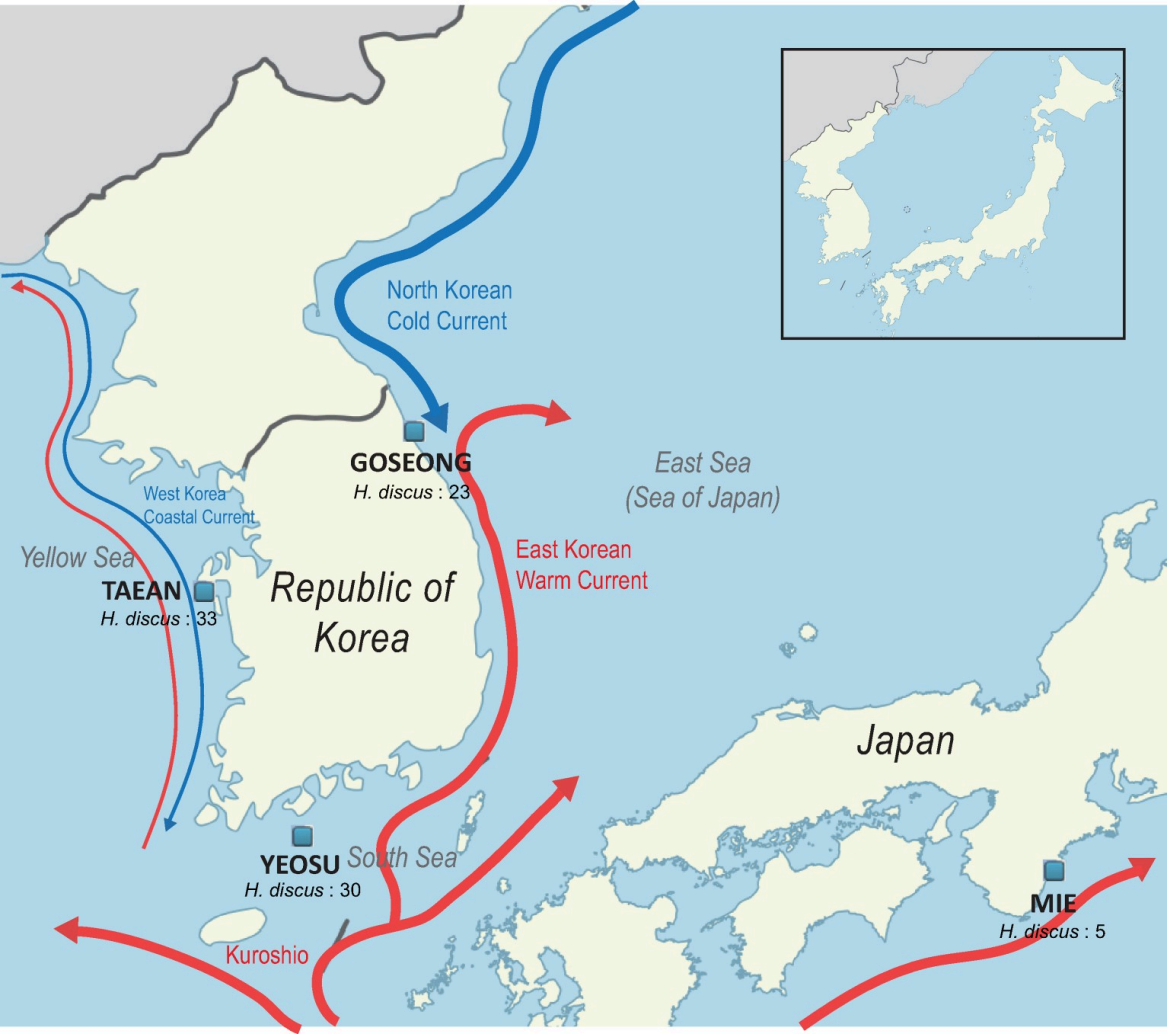
Approximate sampling locations of the abalone populations and trajectories of the major ocean currents. The name, location, and sample size of each population are presented on the map. The major ocean currents around the Korean peninsula are also shown with their names, locations, and the trajectories. Red lines represent warm currents and the blue line represents cold currents.

Five abalone populations (102 samples) were used in this study (Table 1 and Fig 1). Of these, 24 Goseong samples were collected in Goseong, S. Korea, 30 Yeosu samples were collected in Geomundo, Yeosu, S. Korea, and 33 Taean sample were collected in Anmyeondo, Taean, S. Korea. Abalones were obtained by shell fish divers from each regional fishing community, and those released from farms were excluded, based on the green marks that farmed abalones possess on the shell. Five Japan samples from Mie, Japan were provided by Prof. Yamakawa of TUMST, and 11 Red samples collected in California coast, USA were provided by Prof. Lim of Mokpo University. Goseong, Yeosu, Taean, and Mie samples were used for characterization of Korean abalone population, and to identify Korean *H*. *discus hannai*-specific single nucleotide variants (SNVs), Goseong, Yeosu, Taean, and Red samples were used. No permissions were required for sample collection in this study because they were obtained during the season in which regional fishing communities allow harvesting.

**Table 1:** Species, sampling location, sample size, shell length, and sampling date for each abalone population used in this study.

Population	Abalone species	Location	Sample size	Shell length (cm)	Sampling date
**Goseong**	*H*. *discus hannai*	Goseong, S. Korea	24	9±2	July, 2013
**Yeosu**	*H*. *discus hannai*	Geomundo Island, Yeosu, S. Korea	30	10±1	August, 2014
**Taean**	*H*. *discus hannai*	Anmyeondo Island, Taean, S. Korea	33	10±1	August, 2014
**Japan**	*H*. *discus discus*	Mie, Japan	5	11±1	March, 2015
**Red**	*H*. *rufescens*	California, USA	11	-	October, 2015

### GBS library construction and sequencing

Total genomic DNA was extracted from muscle tissue using the DNeasy Blood and Tissue Kit (Qiagen, Hilden, Germany) following the manufacturer’s instruction. The amount of DNA was quantified using the standard procedure of Quant-iT PicoGreen dsDNA Assay Kit (Molecular Probes, Eugene, OR, USA) with Synergy HTX Multi-Mode Reader (Biotek, Winooski, VT, USA) and normalized to 20 ng/μl. DNA (200ng) was digested with 8U of High-fidelity *Pst*I (New England BioLabs, Ipswich, MA, USA) at 37 C for 2 hours and heated to 65°C for 20 minutes to inactivate the enzyme. DNA libraries for genotyping-by-sequencing (GBS) were constructed according to the protocols as described previously [27, 28] with minor modifications. The restriction digestion of DNA with *Pst*I was followed by ligation of adapters with specific barcode for each sample. The sets of 102 ligations were purified using QIAquick PCR Purification Kit (Qiagen). Ligation samples were pooled and 5μl were amplified in 50μl reaction by PCR using AccuPower Pfu PCR Premix (Bioneer, Daejeon, South Korea) and 25 pmol of Illumina adaptors:

5’- AATGATACGGCGACCACCGAGATCTACACTCTTTCCCTACACGACGCTCTTCCGATCT—3’

and

5’- CAAGCAGAAGACGGCATACGAGATCGGTCTCGGCATTCCTGCTGAACCGCTCTTCCGATCT—3’.

PCR cycles consisted of 98°C for 5 min followed by 18 cycles of 98°C for 10 s, 65°C for 5 s, and 72°C for 5 s, with a final extension step at 72°C for 5 min. The PCR product was also purified using QIAquick PCR Purification Kit (Qiagen) and then evaluated the distribution of fragment sizes with BioAnalyzer 2100 (Agilent Technologies, Santa Clara, CA, USA). The GBS library was sequenced in the Illumina NextSeq500 (Illumina, San Diego, CA, USA) with the length of 150 bp single-end reads following the manufacturer instruction. Read count distribution of the GBS library is shown in S1 Fig.

### Variant calling

Before conducting variant calling analysis, we conducted de-multiplexing of GBS data using GBSXtoolkit [29]. Reads of each sample were mapped to the draft genome of *H*.*discus hannai* constructed in our previous study [1], using Bowtie2 [30] with the default options. We then used SAMtools [31] to create index files for reference and bam files. Of the aligned reads, in order to remove possible PCR duplicates, regions covered by abnormally excessive number of identical reads were filtered (in this study, reads with more than 10x of average read depth), since applying PCR duplicates filter is not possible for GBS data utilizing a single-end sequencing approach. Then, genome Analysis Toolkit (GATK) [32] was used for downstream processing and variant calling. Local realignment was conducted using GATK to correct misalignments due to the presence of InDels. The “Haplotypecaller” of GATK was used for calling candidate single nucleotide variants (SNVs) and InDels. To avoid possible false positive variants, argument “VariantFiltration” of the same software was adopted with the following options: 1) SNVs with a phred-scaled quality score of less than 30 were filtered; 2) SNVs with QD (unfiltered depth of non-reference samples; low scores are indicative of false positives and artifacts) <5 were filtered; 3) SNVs with FS (phred-scaled P value using Fisher’s exact test) >200 were filtered, as FS represents variation on either the forward or the reverse strand, which are indicative of false-positive calls; 4) SNVs with MQ0 (the number of reads which have mapping quality zero) >4 or MQ0/DP (proportion of mapping quality zero reads over total depth) >0.1 were filtered to remove uncertain calls; 5) more than 3 SNVs within 10bp window were filtered. Likewise, we also excluded variants that were predicted to overlap with InDel mutation, or overlap with zero coverage regions in more than one sample. SNVs were then further filtered using VCFtools 4.0 [33] for minor allele frequencies < 5%, missing rates > 30%, Hardy-Weinberg equilibrium *P*-values < 0.05.

### PCA, population structure analysis, and population parameters

Principal component analysis (PCA), based on the SNV information of Korea and Japan samples (91 samples), was conducted using SNPRelate R package [34]. For population admixture analysis, VCF file was converted to STRUCTURE input file format using PGDSpider [35], and population admixture was estimated using STRUCTURE [36] under admixture model for K = 1–6 using burn-in length of 5,000 and 50,000 MCMC replicates with 10 iterations. The most likely number of clusters (K) was determined by inferring L(K) and ΔK statistic [37] in STRUCTURE HARVESTER [38]. L(K) is an average of replicated values of LnP(D) at each K. The most likely K is traditionally identified using the highest value of L(K), but in many cases, L(K) continues increasing slightly when the true K is reached. Therefore, we additionally computed ΔK based on Evanno et al [37]. Using ΔK, clearer peak can be shown at the most likely value of K. We utilized pixy [39] for estimating nucleotide diversity in 50Kb sliding windows to take into account invariant sites as well, and pairwise *F*_ST_ values with a 50Kb sliding window for each pair of populations was calculated using VCFtools 4.0 [33]. Lastly, to understand the effects of geographical distance on the genetic distance of populations, we performed Mantel test using GenAlex 6.5 [40] with 10,000 permutations. The genetic distance matrix was generated from 7,000 SNPs randomly selected by data thinning in PLINK 2.0 [41] since GenAlex could not handle whole SNP set. The geographical distance matrix was calculated from minimum distance between each regional group pair.

### SNV annotation and identification of *H*. *discus hannai*-specific SNVs

SnpEff (version 4.2) [42] was used to assign the expected impacts of all the filtered SNVs and their functional annotation. Since there is no database for abalone among the pre-built databases for SnpEff, we used *Haliotis discus hannai* draft genome, constructed in our previous study, and its gene annotation to build database. Korean abalone-specific SNVs were then extracted based on the existence of non-synonymous (missense and nonsense) SNVs present only in Korean abalone populations (a total of 86 individuals) at orthologous positions compared to *H*. *rufescens* species, and in this step, only fixed SNVs were considered. Further, these sets were subjected to enrichment analysis to identify significant SNVs using SnpSift (version 4.2) CaseControl [43]. CaseControl analysis counted the number of genotypes present in case-control groups, and then p-value calculation was performed using Fisher’s exact test to identify SNVs that were significantly associated with case vs. control groups [43]. For Fisher’s exact test, 2 by 2 contingency table was created using two variables, dominant model (Ref (A/A) and Alt (A/a + a/a)) and population information (case and control groups) [43]. Korean abalones were used as the case group, while red abalones were used as the control group, and cutoff p-value < 0.01 was applied.

## Results

### Summary of sequencing reads mapping and SNVs coverage statistics

The resultant *Pst*I GBS library was sequenced using Illumina technology (NextSeq500), and it generated approximately 204.8 million reads from 102 samples which equated to a total of 17.43 Gbp of sequence data (S1 Table). On average, the dataset contained 1,987,914 reads per sample (SD±849,601.2; median: 1,804,068). After quality filtering, the reads were aligned, using Bowtie2, to the draft genome sequence of *H*. *discus hannai* which was constructed in our previous study [1], and the average alignment rate was 72.17% (S1 and S3 Figs). Since digestion by RE is not a random process, GBS can generate a non-uniform distribution of sequenced reads thereby producing biased variant information. Therefore, it is important to evaluate whether data produced by the selected RE can represent the whole genomic range with even spacing. Because not every reads harbor SNV sites, we examined the genome coverage by computing distances between neighboring regions covered by sequenced reads across the reference genome, rather than obtaining distances between SNVs. In each sample, approximately 48,488 covered regions were produced on average, and it means that ideally, the regions were expected to be spaced approximately at 38,749 bp intervals throughout the genome, considering the total length of the reference genome was 1,878,915,344 bp. According to our alignment results, the average distance between the regions was ~33,043bp, which was similar to the ideal distance (S2 Fig). Therefore, we concluded that the desired genome coverage could be obtained on the abalone genome with the GBS library generated by *Pst*I.

After correcting possible misalignments and removal of possible PCR duplicates, SNV calling using GATK pipeline initially detected 529,488 SNVs, and stringent filtering steps finally remained 16,603 SNVs for a set of *H*. *discus* samples (Korea and Japan). The average number of SNVs in each population varied from 3,407 ± 381.8 to 4,186 ± 348.8; the least number of SNV set was observed in the Yeosu abalone population, while the highest value was found in the Japan abalone population (S1 Table). Transitions are the most common type of nucleotide substitutions, and in our final sets of SNVs, 55% of the base changes were transitions and 45% were transversions, with an observed Ti/Tv ratio of 1.19, 1.19, 1.19, and 1.20 for Goseong, Yeosu, Taean, and Japan, respectively (Table 2). For a set of Korea and Red samples, 23,123 SNVs remained after the filtering steps, and all the detected SNVs were functionally annotated and classified into different types of SNV effects using SnpEff (genetic variant annotation and effect prediction tool) (Table 2). Approximately 65.20% and 3.08% of detected SNVs in all five populations were found in intergenic and intron regions, respectively. These SNVs were classified by SnpEff to have modifier impacts, and the prediction of their effects on phenotype was difficult [44]. 1,201 (5.17%) had impacts in exon regions, and among them, 525 were identified to be non-synonymous SNVs. They were annotated as producing moderate to high functional impacts on gene functions by leading to changes in protein effectiveness via altering the coded amino acid sequence and could be associated with the phenotypic differences among the population.

**Table 2:** Summary of all detected SNVs identified from 5 abalone populations.

	Goseong	Yeosu	Taean	Japan	All	Korea	Red	All
**Sample counts**	23	30	33	5	91	86	11	97
**SNV**	16,598	16,603	16,583	15,362	16,603	23,123	14,764	23,123
**Transition**	9,034	9,037	9,026	8,396	9,037	12,470	7,958	12,470
**Transversion**	7,564	7,566	7,557	6,966	7,566	10,653	6,806	10,653
**Ti/Tv**	1.1943	1.1944	1.1944	1.2053	1.1944	1.1706	1.16923	1.1706
**SNV categories**								
** Intergenic**								
** Intergenic region**	14,892	14,897	14,880	13,790	14,897	20,546	12,981	20,546
** Upstream gene variant**	2,214	2,214	2,206	2,070	2,214	3,011	1,946	3,011
** Downstream gene variant**	2,652	2,652	2,650	2,402	2,652	3,571	2,212	3,571
** Intron**	715	715	712	657	715	973	567	973
** Exon**								
** Synonymous variant**	353	353	353	330	353	677	576	677
** Missense variant**	315	315	315	292	315	511	388	511
** Start lost**	1	1	1	1	1	2	1	2
** Stop lost**	0	0	0	0	0	1	1	1
** Stop gained**	10	10	10	8	10	11	3	11
** Stop retained variant**	0	0	0	0	0	1	0	1
** Non coding exon variant**	1,395	1,394	1,391	1,288	1,394	2,176	1,536	2,176
** Splice site acceptor**	1	1	1	1	1	1	0	1
** Splice site donor**	0	0	0	0	0	0	0	0
** Splice site region**	15	15	15	13	15	30	23	30

Functional annotation and locational classification of SNVs are shown. SNV categories are categorized by the types of SNV effects, and each number represents a total number of variants having the effect type.

### Characterization of Korean Pacific abalone populations using SNVs

**Fig 2:**
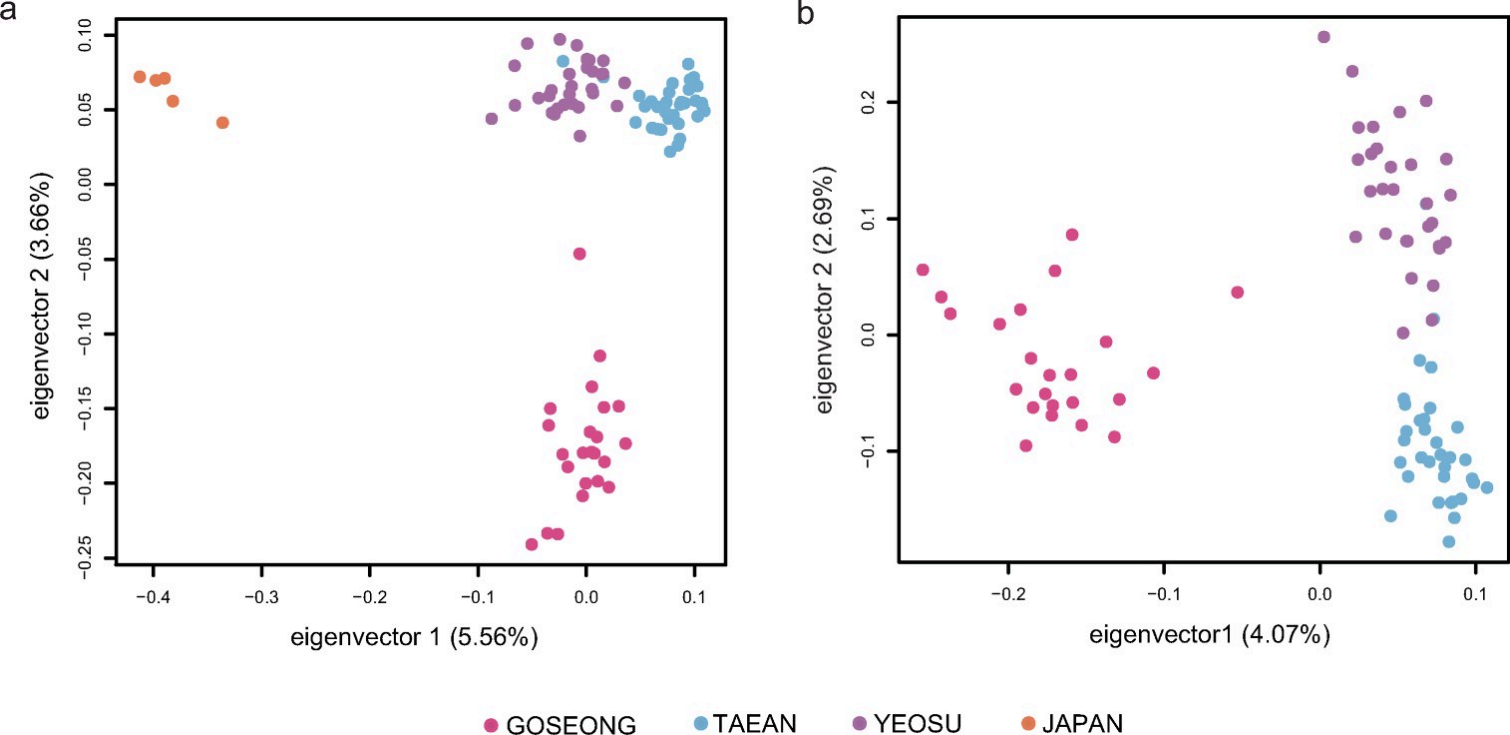
Relationships among Korean *H*. *discus hannai* populations determined by Principal Component Analysis (PCA). PCA of SNV data for 91 abalone samples. 16,603 SNVs with genotypes were used in this analysis. Each dot represents an individual, and each geographic population is represented by different colors. (A) All 91 individuals (Korea and Japan), and (B) 86 individuals (Korea). Korean populations were clustered separately from Japan samples, and among Korean populations, individuals from the Goseong population were clearly separated from the Taean and Yeosu abalones which were clustered close to each other.

**Fig 3:**
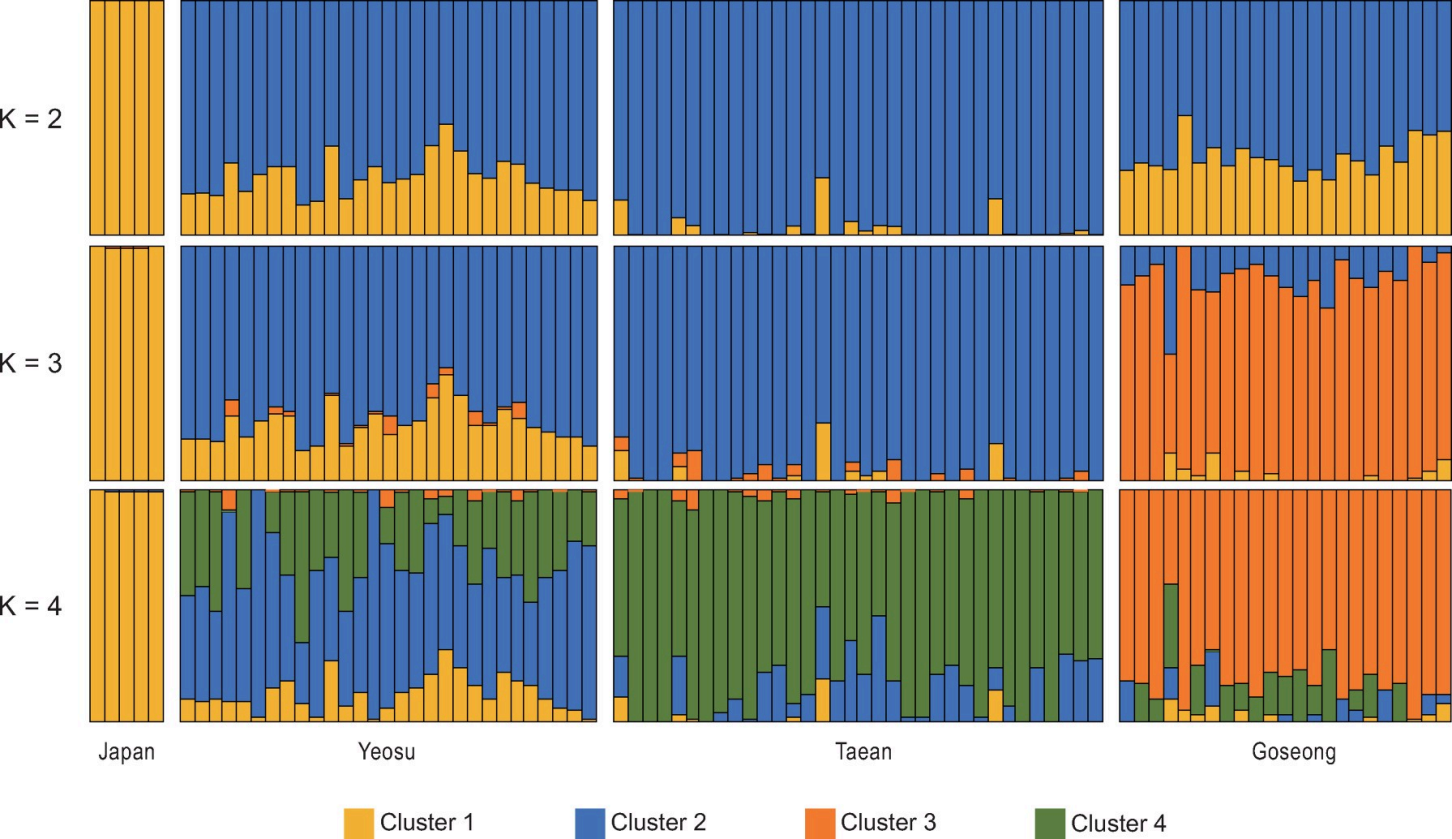
Genetic structures of Korean *H*. *discus hannai* populations. Population structure using the STRUCTURE analysis. Results for K = 2 to 4 are shown. Each vertical line represents an individual, and individuals were grouped by 4 geographic populations. Colors represent the inferred ancestry from K ancestral populations. Populations are labeled below the figure. With a most likely number of K = 3, the Taean population exhibited relatively high population homogeneity, and the Yeosu population showed an admixture of cluster 1 and 2. The Goseong population was the most genetically distinct, with a high proportion of cluster 3.

Several parameters in population genetics were used to characterize the Korean Pacific abalone populations. First, genome-wide measures of nucleotide diversity (pi) on a per-site basis were estimated from the SNV data. Average nucleotide diversity (pi) of three Korean *H*. *discus* populations showed a similar level of genetic diversity (Goseong: 9.906 x 10^−4^, Yeosu: 1.076 x 10^−3^, Taean: 9.686 x 10^−4^), with a relatively low value in Japan population (9.126 x 10^−4^). To investigate the relationships among populations, PCA was performed. Eigenvector 1 clearly separated samples into Korea and Japan group, and eigenvector 2 distinguished Goseong samples (Korea) from Yeosu and Taean samples (Korea) (Fig 2A). To further resolve and separate the individuals from three geographical populations in Korea, additional PCA was performed with those samples. In the resultant scatter plot, the abalone samples of Taean and Yeosu, located on the west and the south coasts of the Korean peninsula, respectively, formed clusters close to each other, and the Goseong abalones were clearly separated from them (Fig 2B). These findings were consistent with STRUCTURE analysis results. The results revealed the presence of three sub-population clusters in our data set as the highest value of L(K) and ΔK appeared for K = 3 (S4 Fig). With K = 3, though the genetic structures of all Korean abalones shared some similarities, strong heterogeneity among geographical groups was shown (Fig 3). Among the groups, it was observed that Taean group exhibited relatively high population homogeneity, with the majority of individuals assigned to a single cluster (cluster 3), in agreement with the fact that Taean is geographically located in a most isolated region, and Yeosu and Goseong populations showed admixtures of cluster 1 and 2 and cluster 2 and 3, respectively. Yeosu population exhibited the highest levels of Japan ancestry, reflecting the possible gene flow. On the other hand, the Goseong samples are the sole constituents of cluster 3, consistent with their distinctive genetic structures identified by PCA. Similar patterns of differentiation among the populations were also presented by the average pairwise *F*_ST_ values. It was identified that the Japan samples were closest to the Yeosu samples (0.1046), followed by Goseong (0.1617) and Taean (0.1888). Among Korean individuals, comparison between Yeosu and Taean population had lower overall *F*_ST_ value compared to the Goseong to Yeosu and Goseong to Taean pairs (Table 3). Similarly, the results of Mantel test detected a significant and slightly positive correlation (Rxy = 0.215, P(Rxy-rand > = Rxy-data) = 0.001, R^2^ = 0.046) between the genetic and geographical distribution (S5 Fig).

**Table 3:** Matrix of pairwise *F*_ST_ values among four abalone populations.

	Japan	Yeosu	Taean
**Yeosu**	0.104555		
**Taean**	0.188840	0.032859	
**Goseong**	0.161680	0.063484	0.072502

Pairwise *F*_ST_ values between each population pair are shown, based on the variant information derived from GBS analysis. The Japan samples were closest to the Yeosu samples, followed by Goseong and Taean. Among Korean individuals, comparison between Yeosu and Taean population had lower overall FST value compared to the Goseong to Yeosu and Goseong to Taean pairs.

### Identification of Korean Pacific abalone-specific SNVs

Additionally, in order to search candidate SNVs accounting for phenotypic differences of Korean abalone populations against red abalones, we further extracted fixed non-synonymous SNVs that were significantly enriched, from 23,123 SNVs for 86 Korean abalone samples and 11 red abalone samples, based on SNV annotation and CaseControl analysis. We identified a total of 809 Korean abalone (*H*. *discus hannai*)-specific SNVs. Among them, 26 SNVs were found within coding regions, from which 23 genes were identified by BLAST search as genes encoding hypothetical protein LOTGIDRAFT_163461, hypothetical protein CGI_10016004, adenosine 3’-phospho 5’-phosphosulfate transporter 1-like isoform X2 exhibited multiple protein mutations (S2 Table). All of the 26 SNVs were missense mutations and include 1 start loss, with no nonsense mutation discovered. We also looked into each SNV on a protein level using InterPro database [45] and investigated whether it caused any physiological and chemical changes (S3 Table). Of the 26 missense mutations, 15 mutations resulted in alteration of chemical properties of amino acids, and five mutations were located in, or in close proximity to, functional domains. Each of the four mutations existed within functional domains were harbored in genes encoding monocarboxylate transporter 12 (major facilitator superfamily domain), zinc finger protein 850-like (zinc finger C_2_H_2_-type), carbonic anhydrase (alpha carbonic anhydrase domain), and chitin binding domain-containing protein (chitin binding domain), and some of them were previously reported to be associated with shell formation of abalones [46–48]. One variant found in close proximity to death domain (DED) was harbored in a gene producing caspase-8 which was involved in innate immune responses [49–51]. We also found the start loss variant was located in von Willebrand factor A (vWA) domain-containing protein 7, which was known to be involved in shell formation as well [47, 52–54].

## Discussion

### Existence of a strong genetic heterogeneity in the Korean Pacific abalones

In contrast to terrestrial species, marine organisms generally show reduced intraspecific genetic differentiation among geographic regions [55–57]. This mainly attributes to the absence of physical barriers to movement in open oceans and their biological characteristics such as large population size and higher dispersal potential during the diverse life-history stages (i.e., planktonic egg, larvae, or adult stages) [58–60]. Marine fishes are generally extensive dispersal, resulting in wide-ranging gene flow due to the active migration of adults and passive dispersal of planktonic larvae [61]. However, marine invertebrates differ from them in which adults have a sedentary lifestyle, and gene flows largely dependents on the passive dispersion facilitated by ocean currents during the planktonic larval phase [4, 60, 62, 63]. Like many sessile mollusks, abalones have strictly benthic habit with poor migratory ability after they first settle, so the primary mode of dispersal is a planktonic larval phase which lasts 4–5 days (in *H*. *discus hannai*) [64–67]. Also, the Korean peninsula is surrounded by three marine ecosystems (East, South, and Yellow sea), each with different biological and oceanographic characteristics [68]. The unique life history of abalones and the characteristic differences of each coastal water around the Korean peninsula render the Pacific abalones as an interesting subject in studying influences of the geographical features and the ocean currents on the population dynamics of marine mollusks.

To assess population differentiation, we investigated patterns of the genetic structure of four regional Pacific abalone populations using 23,123 SNVs, collected from each of the three seas surrounding the Korean peninsula and from coastal waters of Japan. Our results suggested the existence of genetic heterogeneity among Korean groups which was strongly affected by the geographical features of the peninsula and major ocean currents congregating around it. First of all, regression analysis from Mantel test showed that the genetic distance among individuals slightly increased with geographical distance (Rxy = 0.215, P(Rxy-rand ≥ Rxy-data) = 0.001, R^2^ = 0.046), indicating that the geographical distribution contributed partly to the genetic differentiation among the populations (S5 Fig). Also, *F*_ST_, PCA, and STRUCTURE analysis revealed high levels of geographical differentiation and relatively admixed patterns within *H*. *discus hannai* populations around the peninsula compared to Japan samples, and the genetic variation among populations clearly indicated three distinct structures in abalone populations in Korea (Figs 2 and 3). Among them, it was shown that individuals collected from Goseong (East coast), where two different currents meet, were genetically most separated from the other two and were sole constituents of cluster 3 (Figs 1 and 3). Previous studies on *Paralichthys olivaceus* (olive founder) and *Gadus macrocephalus* (Pacific cod) have presented significant differentiation in the East sea areas as well [69, 70], and a similar phenomenon was also observed in admixed population structure of *Donax deltoides* (saltwater clam) at the point of different currents converge [71]. Similar results were also reported in two preceding studies assessed the population genetic structure of Korean wild Pacific abalones. An et al. [19] found different population structures existed between the east populations and the pooled western and southern populations. Park, C.-J. et al. [4] detected the same pattern of genetic separation of the East Sea populations with the existence of similar but distinguishable structures between the South and the West populations, which was observed in this study, too.

At the Korean peninsula, the Kuroshio Current flowing northeastward splits into the Yellow Sea Warm Current and the East Korea Warm Current [68]. The East Korea Warm Current transports marine organisms and a large quantity of heat to the East Sea [72]. This warm current merges with the North Korea Cold Current flowing southward along the Korean coast [68] (Fig 1). The confluence of these currents forms the subpolar front, generally described as located south of 40° N. Therefore, one possible explanation for the origin of cluster 3 in the Goseong population could be the inflow of abalone larvae inhabiting the northern part of the Korean peninsula, along the North Korea Cold Current. No genetic studies have yet been carried out on the abalones of the North Korea region. However, evidence from a report by the Korea Institute for International Economic Policy stated that North Korea exported abalones to China, indicating that the species was distributed north of Goseong as well [73]. In this work, the Goseong population solely represented the East coast population. Additional sampling along the coast will be appreciated to determine the origin of the cluster 3 and better understand the influence of the two currents on the genetic structuring of the East coast abalones. The similar genetic structure between the Taean and Yeosu populations suggested relatively active gene flows among these regions. It was suggested in a previous study that a clockwise gyre consists of a portion of warm current branched off the Kuroshio and then flowing into the Yellow Sea and southward inflow of the West Korea Coastal Current was responsible for the similarity between the two localities, which was responsible for the larval transport [19] (Fig 1). Likewise, it may facilitate the Korean Pacific abalones to mix with larvae dispersed from other geographical populations as the ocean currents congregate around the Korean peninsula from various directions. Also, the existence of at least three distinct genetic structures in Korean Pacific abalones proposed in our study suggests the need for separate management strategies to preserve the genetic diversity of the species.

### Candidate SNVs involved in phenotypically different traits

There are differences in morphological and physiological characteristics between *H*. *discus* and *H*. *rufescens*. The shell length of *H*. *rufescens* can reach up to 31cm, making it the largest abalone species in the world, and the shell length of *H*. *discus* varies between 10cm to 15cm [74]. Also, *H*. *rufescens*’s shell is thicker and stronger, so that it is more resistant to shell-boring ectoparasite infection such as Polydora spp. than *H*. *discus* species [3, 75]. Due to these evident differences, we utilized the SNV sets of pooled Korean *H*. *discus* populations and *H*. *rufescens* population to search for candidate SNVs potentially associated with the phenotype differences. For this analysis, we used only fixed non-synonymous SNVs with moderate to high impacts on gene function, and we were able to discover 26 SNVs existing in coding regions which were significantly enriched in the case-control analysis. Some of these SNVs were located in shell formation related genes such as vWA domain-containing protein 7, Kunitz-like protease inhibitor, carbonic anhydrase alpha, and chitin binding domain-containing gene [46–48, 52–54, 76], and for latter two genes, the SNVs were located within functional domains. It also enabled us to test the capability of GBS SNV datasets to detect population-specific SNVs which were potentially implicated with specific traits.

The formation of the molluskan shell is regulated by a matrix of extracellular macromolecules that are secreted by the shell-forming tissue, mantle [76]. This matrix is a complex mixture of CaCO_3_, shell matrix proteins (SMP), pigments, lipids, polysaccharides, and glycoproteins [46, 76]. The molecular mechanisms of this calcifying shell formation are only beginning to be elucidated in Mollusks, but it was reported in many studies that the SMPs play important roles in shell formation and forming distinct morphology [46, 77]. vWA, carbonic anhydrase and chitin-binding domain-containing proteins are well known SMPs in many marine mollusk species. vWA domains in extracellular proteins mediate adhesion via metal ion-dependent adhesion sites, implying its role in protein-protein interaction between layers [47]. They are found in various marine molluskan species, including *Crassostrea gigas*, *Mytilus coruscus*, *Lottia gigantea*, *Pteriida fucata*, etc. [47, 52–54]. Carbonic anhydrases are responsible for controlling pH by converting CO_2_ to HCO_3_^-^ in Nacrein [47]. And chitin-binding domain plays significant roles in the formation of biominerals because chitin is the major framework in which CaCO_3_ forms. It is usually found together with vWA in SMPs, and both are typically domains of collagen, the fundamental component of ECM [46, 48]. Kunitz-like protease inhibitor was previously observed in the shell matrix of *Haliotis asinina* and also found from the mantle cells of diverse mollusks [76]. The most likely function of these inhibitors was suggested as the protection against degradation by exopeptidases which were produced by marine microorganisms [76] such as tissue inhibitors of metalloproteinase identified in *P*. *fucata* species [47]. However, no studies have identified genes directly responsible for molluskan shell size or thickness yet, and the 26 Korean *H*. *discus hannai*-specific SNVs presented here are the first to be reported in this study. Moreover, since the present study had a limitation in a relatively small sample size of *H*. *rufescens* population, and thus they were hard to represent the genetic characteristics that covered species’ range, future studies including a higher number of samples are needed to further validate these preliminary findings. Also, most population-specific genetic differences are likely to be neutral, and it is above the capability of this study to determine how each SNV functionally affects the gene. Thus, we cautiously suggest these identified SNV sets as promising candidates to guide further investigation.

## Conclusions

This study evaluated the genetic diversity and the population structure of *H*. *discus* populations around the Korean peninsula and elucidated the impacts of the geographical features and the ocean currents in the gene flow among marine mollusk populations. Based on the patterns of population genetic structuring in the *F*_ST_, PCA, and STRUCTURE, the ocean currents congregating around the Korean peninsula were key factors of genetic heterogeneity among groups as the directionalities of ocean currents worked as a directional bias in gene flow. Besides, by comparison of variant sets, we identified Korean Pacific abalone-specific variants, which might be associated with phenotypically different traits between groups. Our results will provide valuable data for the genetic conservation and management of wild abalone populations in Korea and help the future GBS studies on the marine mollusks.

## Supporting information

S1 Fig De-multiplexed read count distribution of 102 GBS libraries of Haliotidae population. (TIF)

S2 Fig Distribution of distances between regions covered by sequenced reads across the abalone genome. (TIF)

S3 Fig Regional distribution of whole genome variants of 102 *Haliotis* samples using SnpEff. (TIF)

S4 Fig Diagram of Mean L(K) (±SD) and Delta K (ΔK) in STRUCTURE analysis. (a) Mean L(K) (±SD) over seven runs from K = 1 to K = 6; (b) Delta K (ΔK); (c) Estimation following Evanno et al.
(TIF)

S5 Fig Correlation between geographical distances and genetic distances among 86 Korean *H*. *discus* samples based on Mantel test. (TIF)

S1 Table Summary of sequencing results and SNVs from the 102 abalone samples. (XLSX)

S2 Table Functional description of genes harboring 26 *H*. *discus*-specific SNVs identified by BLAST. (XLSX)

S3 Table List of 26 *H*. *discus*-specific SNVs including coding effect, codon/amino acid change, and property change information. (XLSX)

S1 Appendix Final set of SNVs after filtering steps in VCF (variant call format) file. (GZ)
