# Supplementary figures and images for: Genotyping-by-Sequencing of the regional Pacific abalone (Haliotis discus) genomes reveals population structures and patterns of gene flow
Source: PLoS One. 2021 Apr 7;16(4):e0247815. doi: 10.1371/journal.pone.0247815 (PMC8026068; doi:10.1371/journal.pone.0247815)

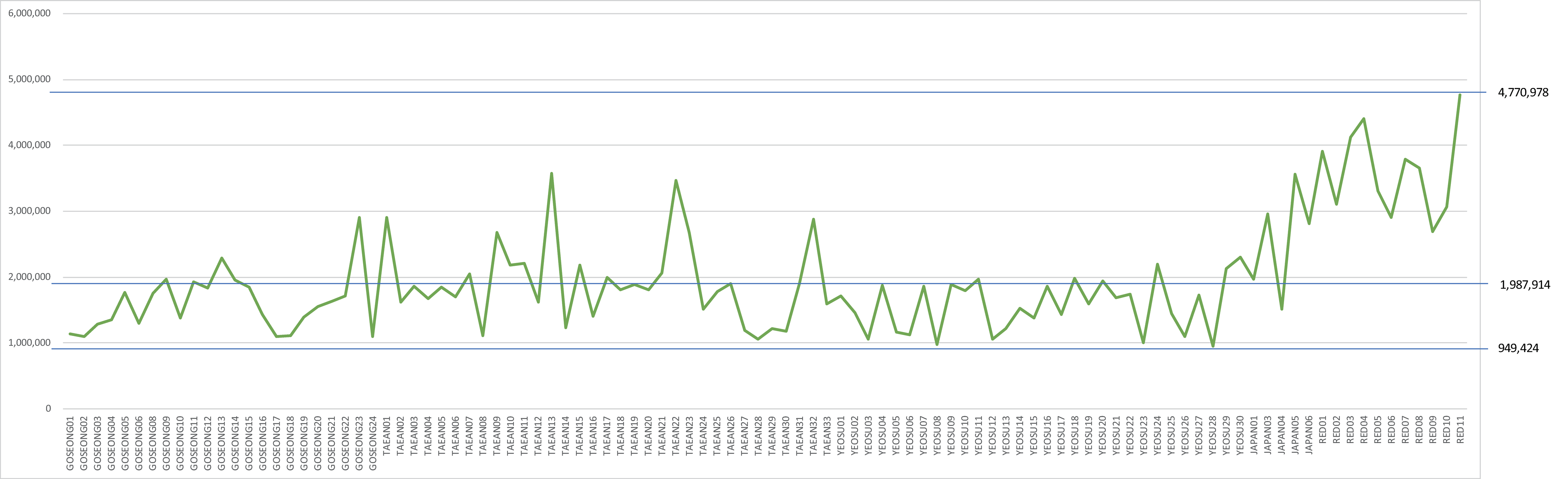

Supplement: S1 Fig — (TIF) [file pone.0247815.s001.tif]

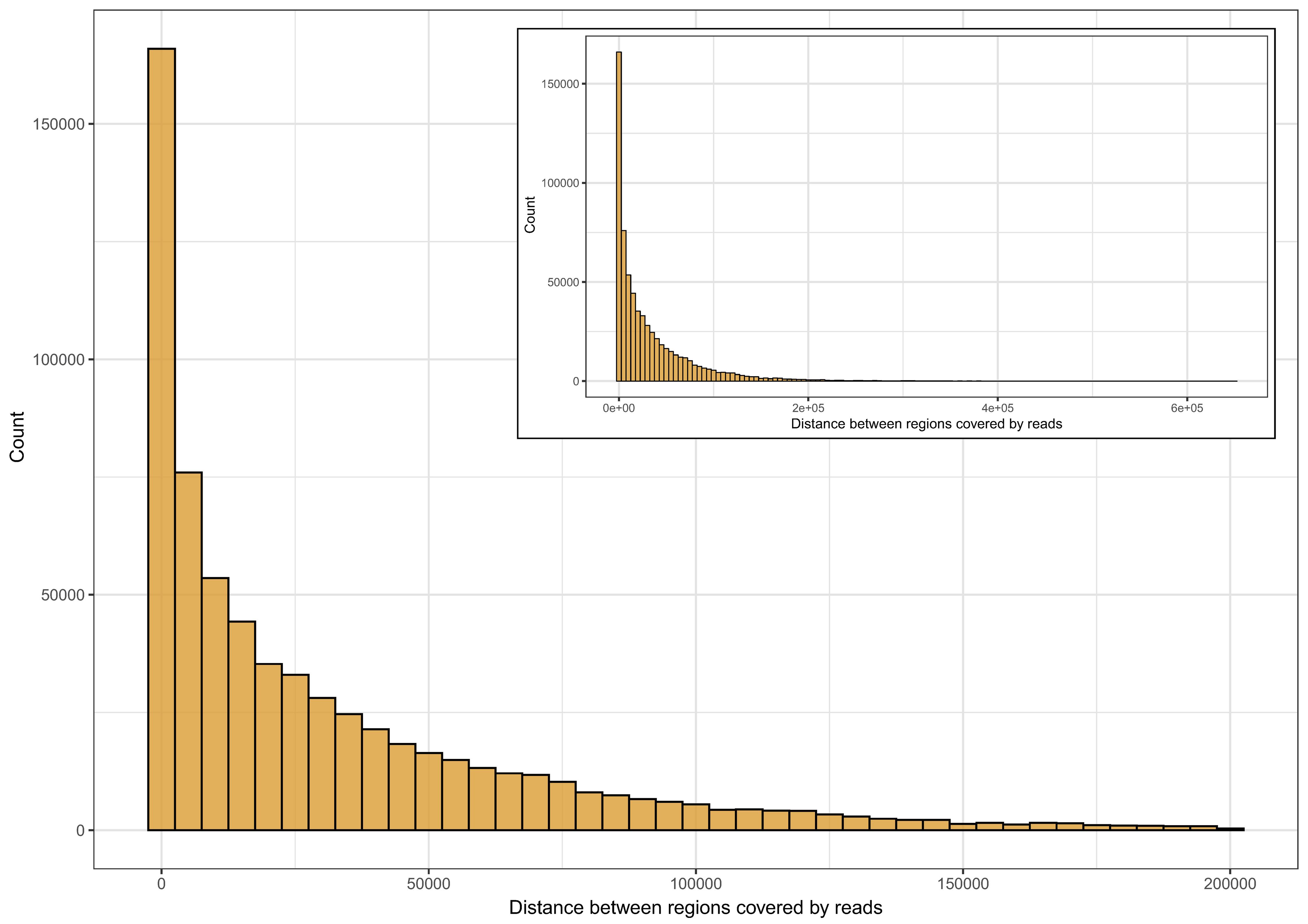

Supplement: S2 Fig — (TIF) [file pone.0247815.s002.tif]

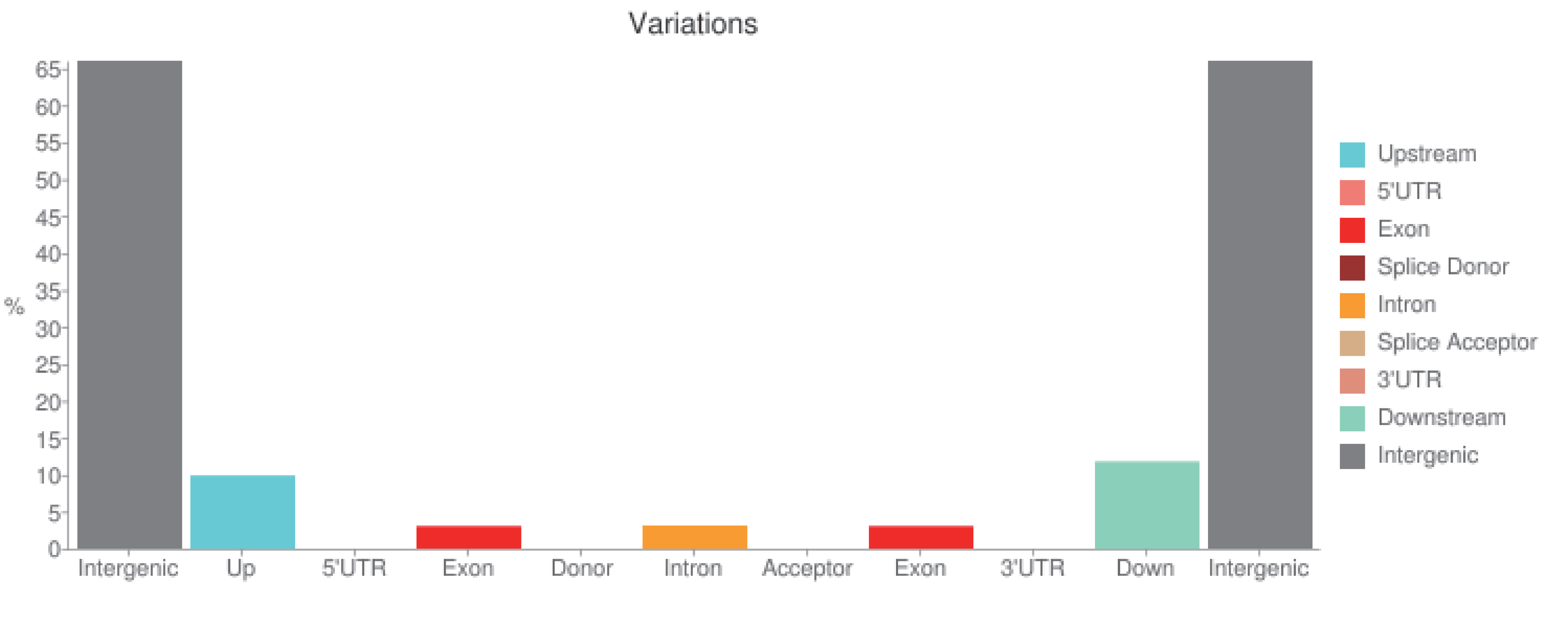

Supplement: S3 Fig — (TIF) [file pone.0247815.s003.tif]

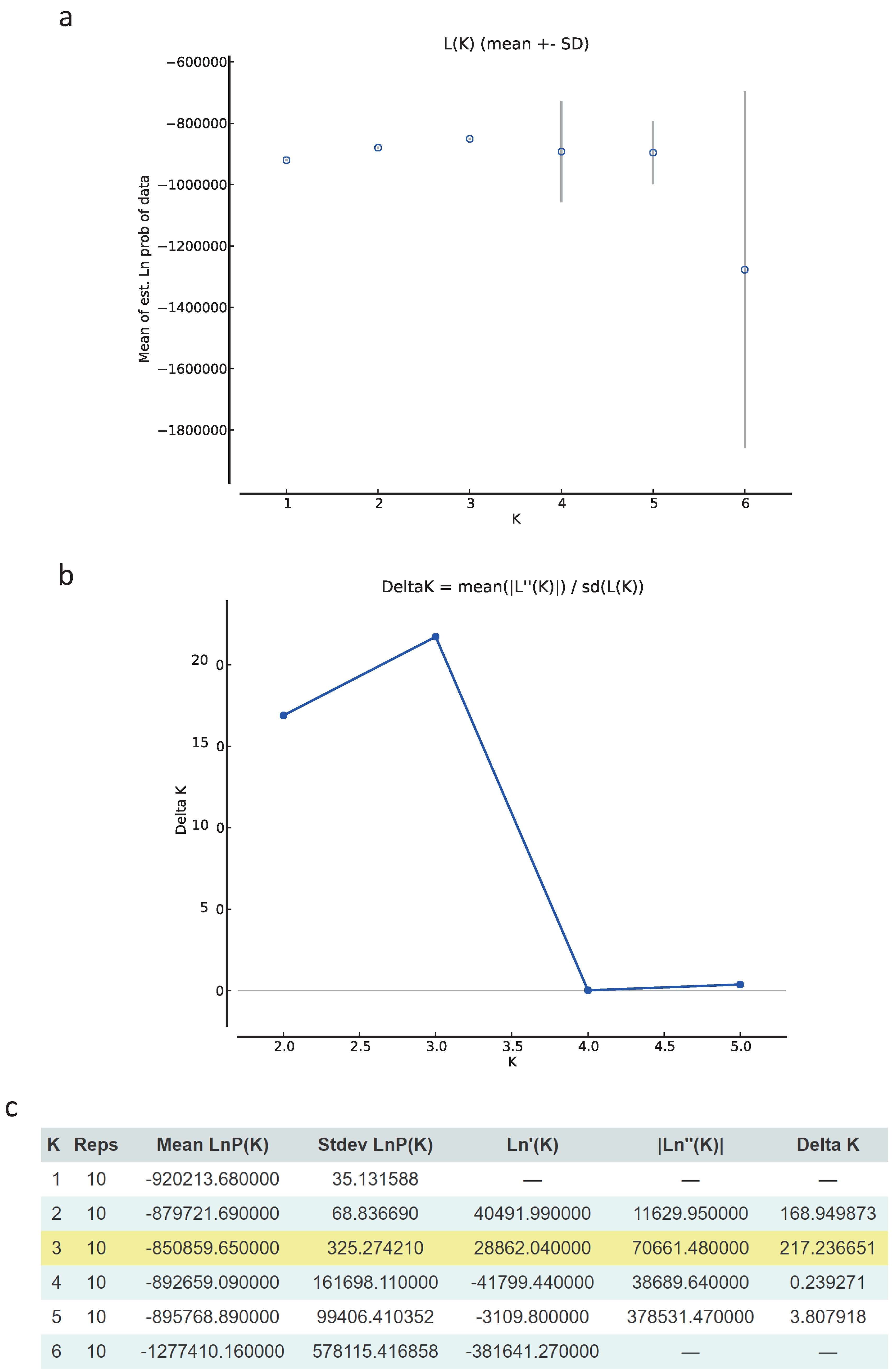

Supplement: S4 Fig — (a) Mean L(K) (±SD) over seven runs from K = 1 to K = 6; (b) Delta K (ΔK); (c) Estimation following Evanno et al. (TIF) [file pone.0247815.s004.tif]

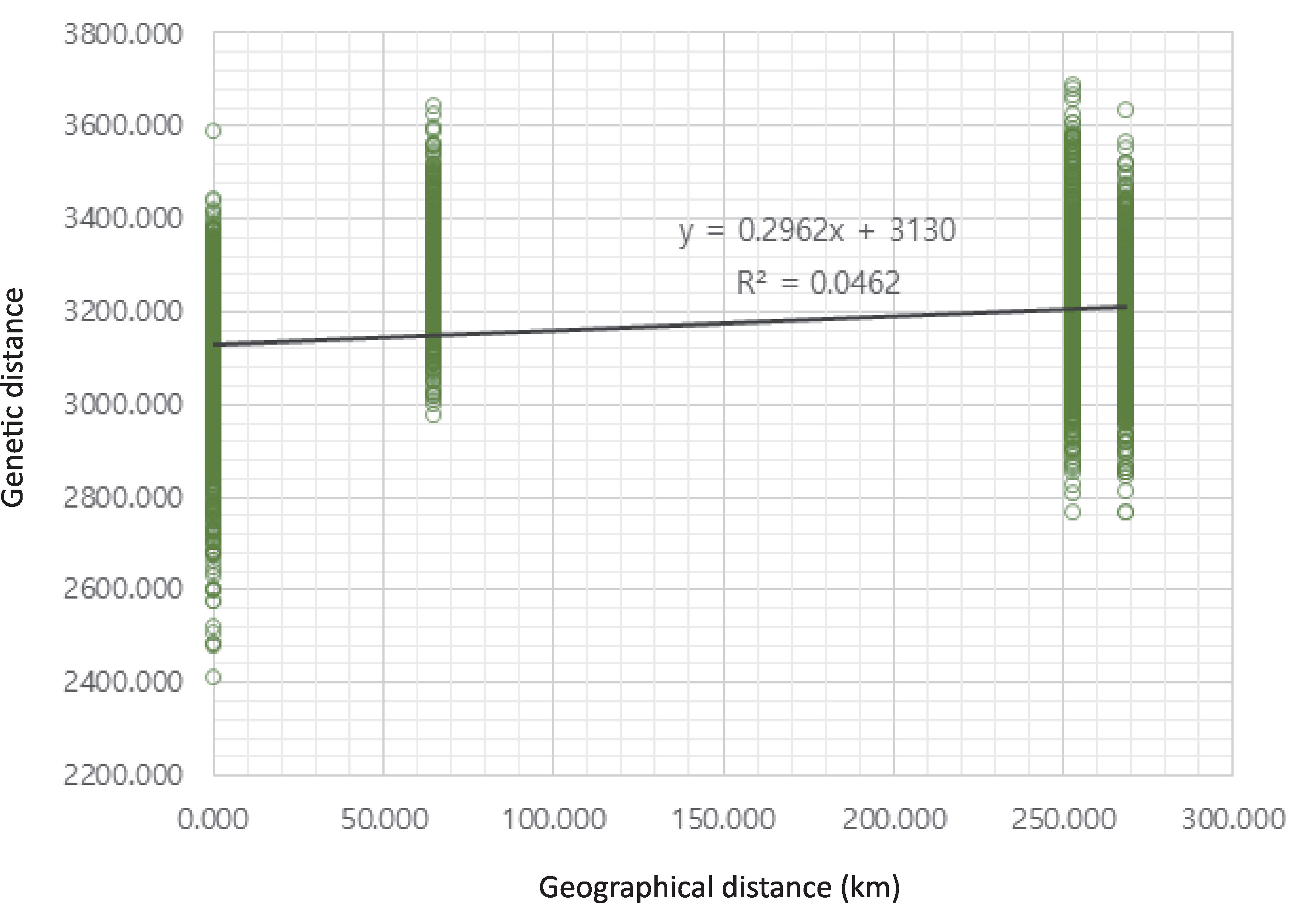

Supplement: S5 Fig — (TIF) [file pone.0247815.s005.tif]
